# Enrichment of *Candida* associated with dysbiosis contributes to mucosal CD4^+^FOXP3^+^ regulatory T cell accrual and their dysfunction in aging

**DOI:** 10.3389/fimmu.2026.1714595

**Published:** 2026-03-19

**Authors:** S. Jayaraman, S. S. Mahalingam, Z. Zhu, F. Faddoul, A. Paes da Silva, R. Asaad, N. Bhaskaran, E. Schneider, T. Taylor, S. Horne, A. Yoo, L. Zhang, A. Burgener, Pushpa Pandiyan

**Affiliations:** 1 Department of Biological Sciences, School of Dental Medicine, Case Western Reserve University, Cleveland, OH, United States; 2 Department of Population & Quantitative Health Sciences, School of Medicine, Case Western Reserve University, Cleveland, OH, United States; 3 Advanced Education in General Dentistry, School of Dental Medicine, Case Western Reserve University, Cleveland, OH, United States; 4 Department of Periodontics, School of Dental Medicine, Case Western Reserve University, Cleveland, OH, United States; 5 University Hospitals Cleveland Medical Center AIDS Clinical Trials Unit, Cleveland, OH, United States; 6 Center for Global Health and Diseases, School of Medicine, Case Western Reserve University, Cleveland, OH, United States; 7 Department of Obstetrics, Gynecology and Reproductive Sciences, University of Manitoba, Winnipeg, MB, Canada; 8 Unit of Infectious Diseases, Department of Medicine Solna, Center for Molecular Medicine, Karolinska Institute, Karolinska University Hospital, Stockholm, Sweden; 9 Department of Pathology, School of Medicine, Cleveland, OH, United States; 10 Department of Molecular Biology and Microbiology, School of Medicine, Cleveland, OH, United States; 11 Center for AIDS Research, Case Western Reserve University, Cleveland, OH, United States

**Keywords:** Candida, inflammaging, mucosal immunity, oral microbiome, Tregs (Regulatory T cells)

## Abstract

Age-associated T cell dysfunction is a defining feature of inflammaging and immunosenescence, the progressive decline in immune competence observed with advancing age. Here we identified the association between aging (defined as age >60) and fungal dysbiosis, notably characterized by increased colonization of *Candida* species in the oral mucosa. There is also a notable enrichment of other taxa related to the order Saccharomycetales in older individuals. In contrast, younger individuals exhibit a greater abundance of *Cryptococcus*, *Yarrowia*, *Kluyveromyces*, and various Incertae sedis lineages. Further analysis, stratified by HIV status, shows that older individuals in both healthy and HIV+ groups display significantly higher levels of *Candida.* Gingival tissues reveal that both healthy older group and HIV-positive group exhibit elevated levels of CD4^+^FOXP3^+^ regulatory T cells (T_regs_) along with increased salivary concentrations of soluble TLR-2 and IL-6 compared to younger healthy group. Importantly, the abundance of *Candida* is positively correlated with elevated levels of mucosal T_regs_, dysfunctional T_regs_ (T_regDys_), and hyperactivated CD4^+^ T cells. *In vitro* experiments provided mechanistic insights by further demonstrating that *Candida* can induce both proliferation and dysfunction of T_regs_ in an IL-6 dependent manner, supporting the notion that *Candida* plays a role in oral T cell senescence and inflammaging. Collectively, these findings underscore a direct relationship between the commensal mycobiome and T_reg_ population, which normally promotes mucosal homeostasis but becomes susceptible to dysfunction with aging.

## Introduction

The gradual decline in immune function, typically commencing midlife (~60 years onward) significantly contributes to the heightened susceptibility to infectious diseases and malignancies, which are among the leading causes of morbidity and mortality in the elderly population (1–4). Clinical consequences of T cell senescence become more apparent after thymic involution, when peripheral expansion and homeostatic proliferation of existing T cells can no longer compensate. While certain studies suggest that the gut microbiome remains relatively stable with chronological aging, other research indicates a reduction in microbial diversity as age advances (5–7). There is no specific chronological threshold at which the microbiota composition changes abruptly; rather, these shifts occur gradually over time (5–7). Although some of microbiome changes may be particularly associated with immune health changes, the causative and mechanistic details are unclear.

Resident commensal fungi, the mycobiome, plays an active role in shaping intestinal health and disease (8, 9). Dysbiosis of fungi, characterized by reduced diversity and an altered balance of fungal taxa is linked to inflammatory bowel diseases (IBD), Crohn’s disease (CD), liver disease, metabolic syndromes, and infections (8, 9). While the oral cavity is among the initial mucosal sites identified for asymptomatic fungal carriage (10, 11), oral mycobiome studies in the context of aging have shown varied results in different cohorts (12). Indeed, aging is known to alter oral mycobiome and is associated with hormonal changes, oral candidiasis, Xerostomia, oral caries and inflammation (13–15). We and others have previously shown that aging is related to oral inflammation and regulatory T cell population (T_reg_) dysfunction during *Candida* infection in mice (1, 2, 16–19). *Candida* sp, predominantly a asymptomatic fungal colonizer of mucosal surfaces, also shows a higher prevalence in patients with compromised mucosal barriers (19–22), and is strongly associated with oral thrush and poor oral immune and systemic health (13, 23, 24). However, whether shifts in microbial components within the microbiome or chronic infections directly drive T-cell dysregulation during aging (16, 19, 25–30), or whether T-cell dysregulation itself alters the microbiome (16–19), remains unclear. This is one of the key questions we have addressed in the context of T_regs_ and mycobiome in the current study. Here, we sought to determine how aging modulates the changes in resident oral fungi and oral mucosal immune system. Using ITS sequencing of saliva samples and flow cytometry of gingival mucosal T cells in young and aged individuals, and mechanistic studies in tonsil organoids, our data reveal the impact of aging in mycobiome composition leading to direct changes in T_regs_ in the mucosa. Our results suggest an alteration in the structure of the oral mycobiome, favoring increased opportunistic colonization by *Candida* and Saccharomycetes in the older group. These findings show a novel and complex interplay between fungal dysbiosis and aging-dependent immune system changes, which may contribute to the overall inflammatory burden by inducing a qualitative impairment of key immune cells in oral mucosa.

## Results

### Oral mycobiome is dominated by *Candida* in oral mucosa

**Figure 1:**
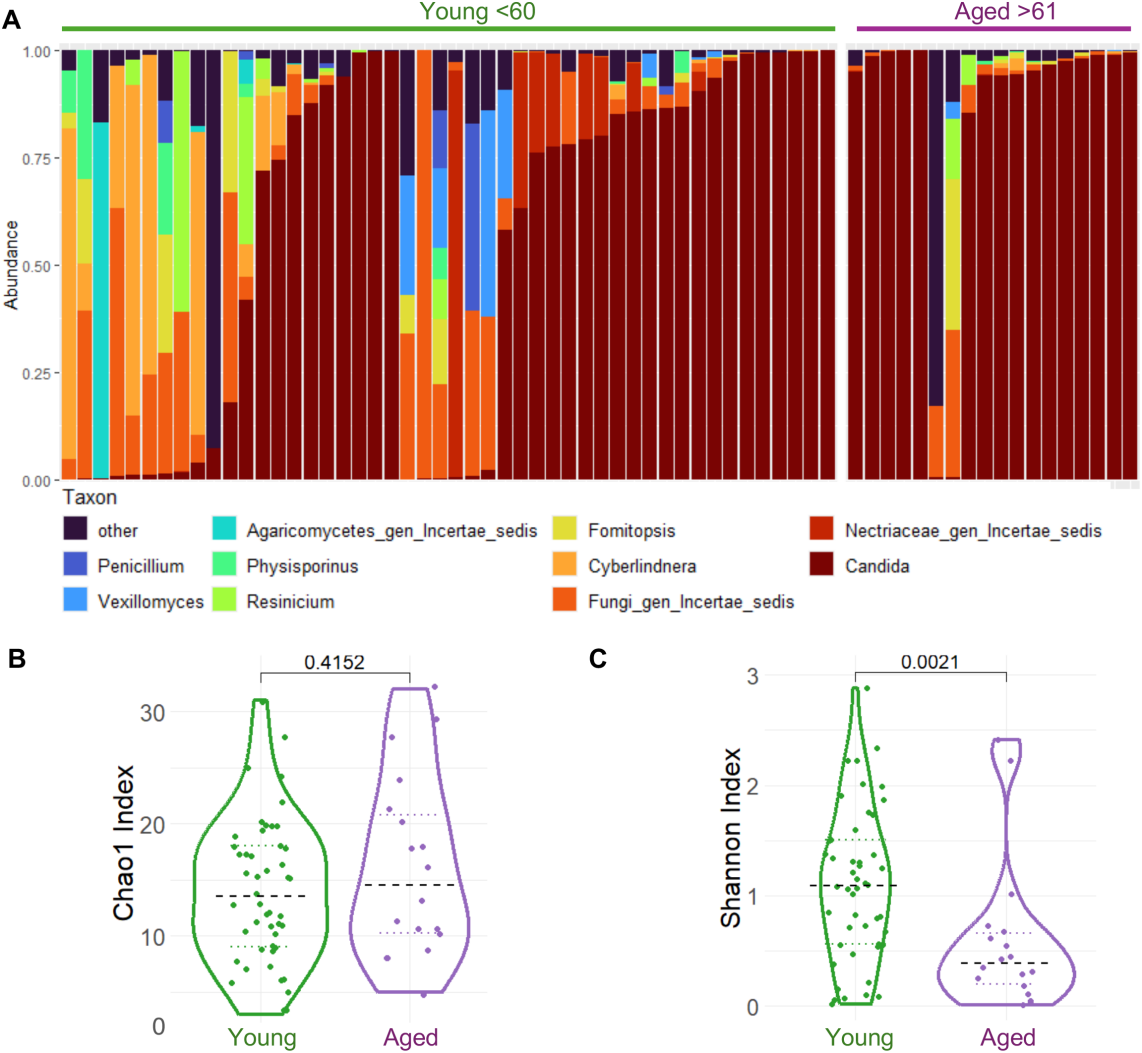
Oral Mycobiome analysis comparing young versus aged individuals. Saliva samples (n=66) were processed for ITS (mycobiome) sequencing (Young; n=48 and Aged; n=18) . UNITE reference database used to train a Naive Bayes classifier and taxonomically classify filtered reads into ASV with 99% phylogenetic similarity. **(A)** Stacked bar plot showing the overall distribution and relative abundance of fungi (showing top 10 taxa by mean relative abundance) in saliva samples obtained from participants (n=66) Taxa grouped under "Other" represent genera outside the top 10. **(B)** Violin plot comparing the Chao1 richness index between older and younger participants. **(C)** Violin plot of the Shannon diversity index in the same age groups. While species richness was comparable (p=0.4152). Shannon diversity was significantly lower in aged individuals (p=0.0021) indicating lower evenness taxonomic complexity in their oral mycobiome communities.

The integrity and composition of the resident microbiota, which includes fungal communities, are crucial for the maintenance and proper functioning of mucosal immune environment. Given that many of the previous aging microbiome studies have primarily focused on individuals over 65 years old, a group already prone to frailty and systemic inflammation, both of which can influence the microbiome, we stratified our cohort by age in individuals with healthy oral gingiva and no evidence of inflammation (See **Supplementary Table 1**, **Supplementary Materials** and **Supplementary Methods** for inclusion and exclusion criteria). For a comparative analysis in a group with known alterations in oral T cell dysregulation, we included HIV-positive individuals from our previous cohorts (see **Supplementary Table 1**, **Supplementary Materials** and **Supplementary Methods** for information on CD4 counts) (11, 29–31). We have previously shown that HIV+ individuals within the same cohort have elevated levels of T_regs_ and dysfunctional T_regs_ in oral mucosa (28–30). We analyzed the composition of oral mycobiome by comparing saliva samples from young and aged individuals (n = 66), using ITS-based sequencing. The sequencing runs generated 2989567 total reads, of which 100% contained identifiable tags. On average, each sample yielded 35847 sequences with a mean read length of 251 bases. The results demonstrate that the observed mycobiome aligned with previously established profiles (32). A stacked bar plot illustrated the overall distribution and relative abundance of fungi, highlighting the top 10 taxa by mean relative abundance in saliva samples collected from the participants (**Figure 1A**). Taxa categorized under “Other” included genera outside the top 10. The most common genera detected included *Candida, Cyberlindnera, Penicillium*, and members of the order Saccharomycetales. Notably, a considerable proportion of fungi in many individuals were classified as *incertae sedis*, reflecting taxa of uncertain phylogenetic placement.

We then utilized Principal Coordinates Analysis (PCoA) based on weighted UniFrac distance to explore age-related differences in oral fungal beta diversity (**Supplementary Figure 1**). The results illustrated ellipses for both the older group (depicted in purple) and the younger group (depicted in green) with 95% confidence. The analysis revealed that the first principal coordinate (PC1) and the second principal coordinate (PC2) account for 48.86% and 8.24% of the total variance, respectively. This suggests that these two dimensions captured a significant portion of the variability in our data. Although the PERMANOVA test yielded barely statistically significant results (p = 0.051), we observed a broader dispersion of data points among the younger individuals. This wider spread might indicate greater inter-individual variability in the mycobiome composition of the younger group, suggesting that their fungal communities are more diverse compared to those of the older group. Violin plots showed Chao1 richness and the Shannon diversity indices comparison between older and younger participants. The analysis revealed that while species richness was similar across age groups (p = 0.4152) (**Figure 1B**), the Shannon diversity index was significantly lower in aged individuals (p = 0.0021) (**Figure 1C**). These data suggest that younger participants possess greater evenness and taxonomic complexity in their oral mycobiome communities.

### Enrichment of *Candida* in older individuals

**Figure 2:**
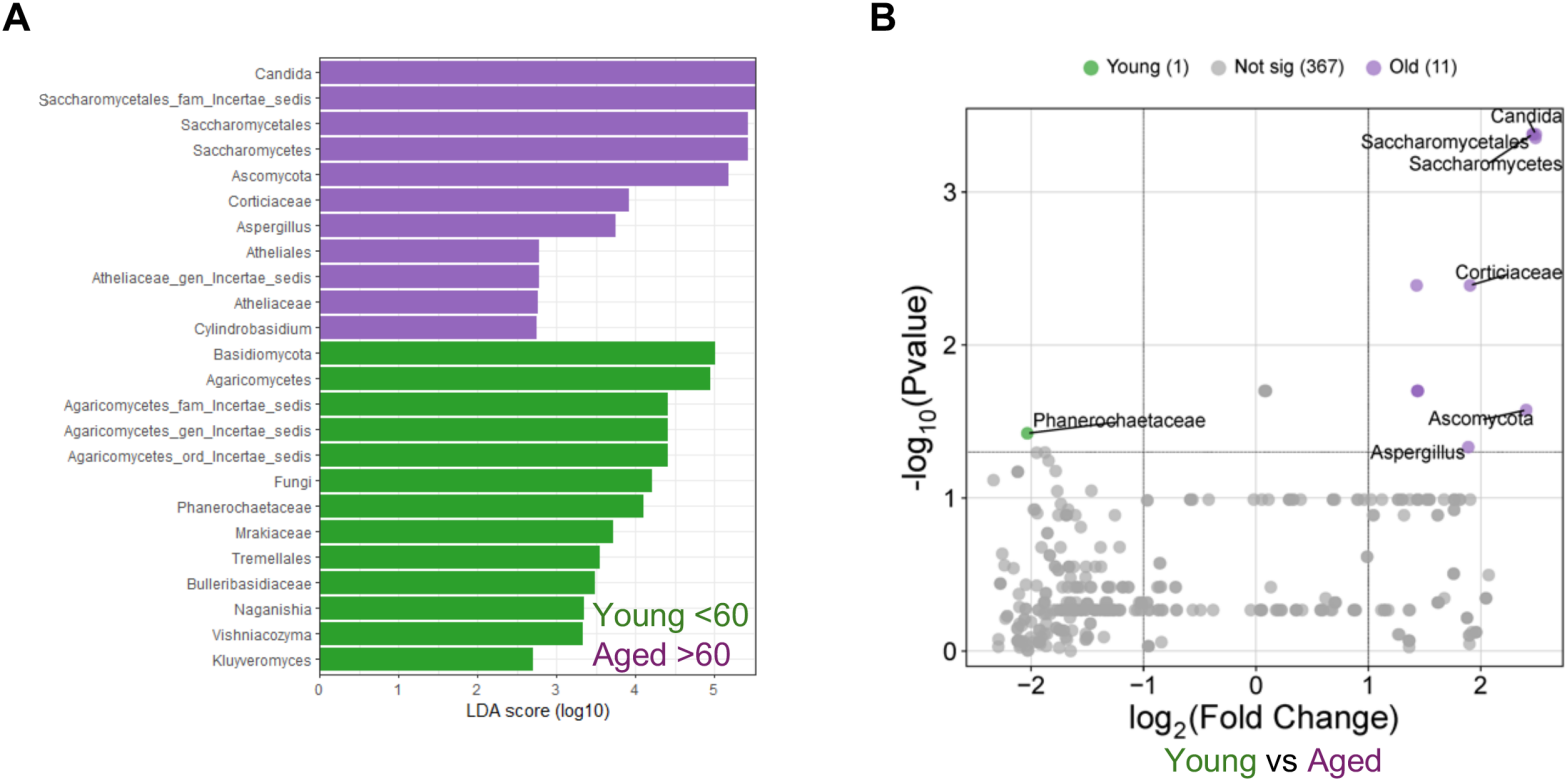
Oral Mycobiome analysis showing enrichment of *Candida*, *Saccharomycetes*, and *Aspergillus* in aged individuals. Saliva samples (n=66) were processed for ITS (mycobiome) sequencing (Young; n=48 and Aged; n=18). UNITE reference database was used to train a Naive Bayes classifier and taxonomically classify filtered reads into ASV with 99% phylogenetic similarity. **(A)** LDA score; Linear discriminant analysis effect size (LEfSe) was used to identify age-associated fungal taxa enriched in younger (green) and older (purple) participants. Differential features were determined using Kruskal-Wallis and pairwise Wilcoxon tests (p<0.05), with a minimum LDA score of 2 (logo scale). **(B)** Volcano plot displaying differential abundance of taxa between young and old individuals. The x-axis indicates the log_2_ fold change (Young vs Aged), and the y-axis represents −log_10_ p-values. Young (1) and Old (11) indicate the number of significant hits in the respective groups and Not Sig (367) denotes the hits that were non-significant between the groups. Statistically significant taxa enriched in the younger (green) and older (purple) groups are highlighted.

Next, we examined age-associated differences in specific fungal communities of younger and older individuals. Using Linear Discriminant Analysis Effect Size (LEfSe), we observed specific fungal taxa enriched in each age group, as illustrated by the LDA scores (**Figure 2A**). Saliva samples from older individuals (purple) exhibited a significant enrichment of *Candida* and other taxa related to Saccharomycetales. In contrast, younger individuals (green) showed enrichment for *Cryptococcus*, *Yarrowia*, *Kluyveromyces*, and various *Incertae sedis* lineages. This suggests an age-associated shift in the oral mycobiome structure, indicating a tendency towards increased opportunistic colonization in older individuals. The volcano plot further illustrated these differences by displaying the differential abundance of fungal taxa between young and old individuals (**Figure 2B**). The results suggest that *Phanerochaetaceae* is the only taxon associated with younger individuals, whereas older group had an abundance of multiple taxa, with *Candida* being the most significant hit. Overall, a significant shift towards *Candida* dominance was observed in the oral mycobiome of elderly individuals. This distribution pattern highlights the potential for increased opportunistic colonization in the aging population, with *Candida* playing a prominent role in the mycobiome of older individuals.

### Changes in oral mucosal T_regs_ and inflammatory microbial ligands during aging and HIV infection

**Figure 3:**
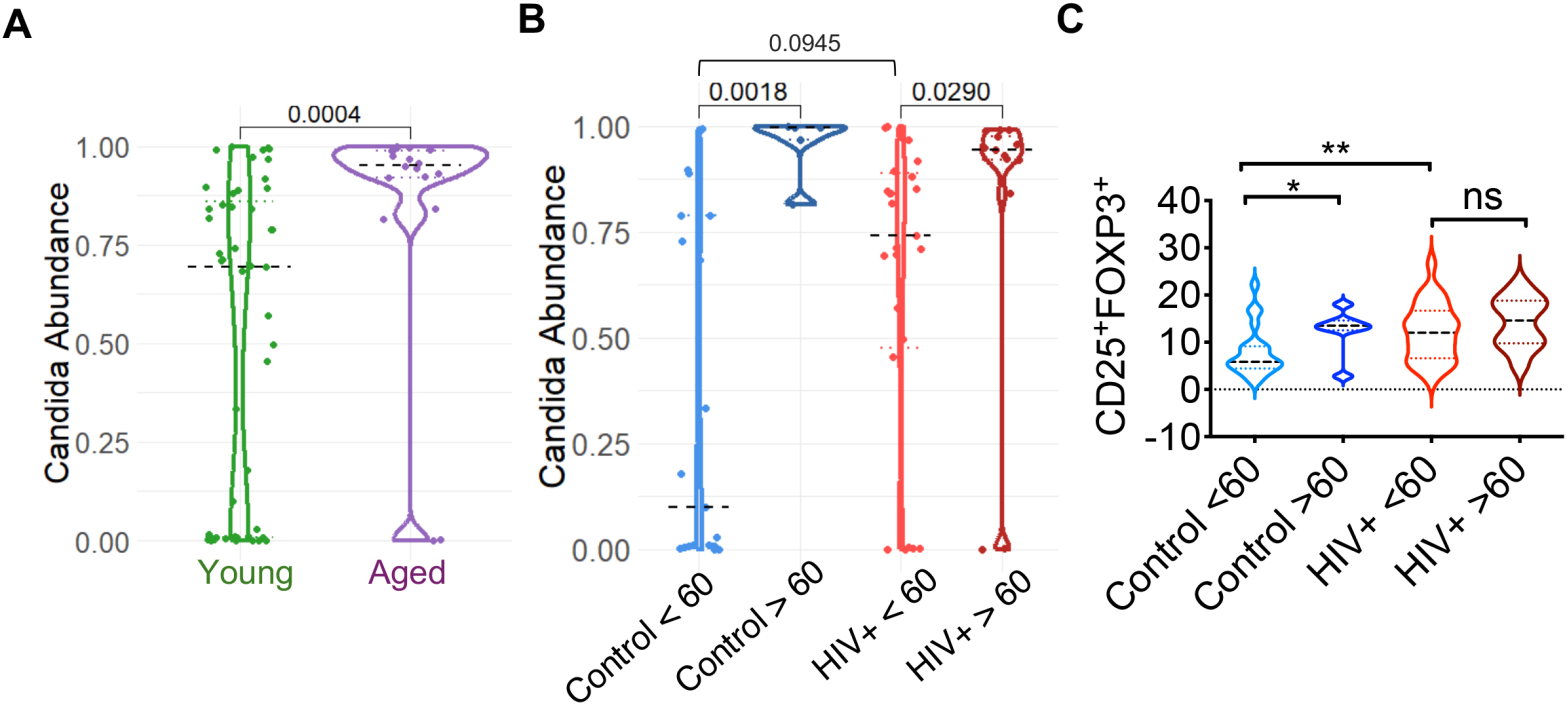
Comparison of *Candida* relative abundance and gingival   CD4^+^CD25^+^FOXP3^+^_Tregs_ across groups stratified by age and HIV status. **(A)** Violin plot showing the relative abundance of *Candida* in young (age<60) and aged (age >60) participants. Each point represents an individual sample. The Wilcoxon rank-sum test revealed a statistically significant increase in Candida abundance in the aged group (age >60) (p = 0.0004). **(B)** *Candida* relative abundance comparing young and aged individuals in healthy control and HIV+ subgroups, showing *Candida* abundance in the older individuals in both subgroups. **(C)** Oral gingival T_reg_ proportions, as assessed by flow cytometry, comparing young and aged individuals in healthy control and HIV+ subgroups. ns, non significant; * P<0.05, ** <0.005.

Informed by prior data on immune dysfunction in individuals over 60 and those with HIV infection (HIV+) (28, 29), here we conducted detailed analyses by grouping participants according to age (age<60; Young and age >61; Aged) and further stratifying them based on HIV status. Previous reports have shown that the fungal composition of inflamed mucosa is predominantly characterized by an increased abundance of *Candida* spp (8, 9). Accordingly, we aimed to investigate inflammatory markers in the context of *Candida* enrichment. Because T cell dysfunction and enrichment of inflammatory pathways observed in oral mucosa of HIV^+^ individuals (28–30), we sub-stratified the two groups based on HIV positivity and compared the mycobiome composition in these subgroups. As expected, increased relative abundance of *Candida* was observed in aged group in the absence of stratification (p-value; 0.0004) (**Figure 3A**).

Increased *Candida* abundance was also observed in older subgroups of both healthy control and HIV+ groups (**Figure 3B**). However, *Candida* levels were comparable between the control and HIV+ subgroups (**Figure 3B**). This analysis highlights differences in *Candida* prevalence and the potential effect of age and immune health in fungal colonization. To further identify the potential association of aging and mycobiome with changes in bacteriome, we also conducted 16S rRNA sequencing of the samples in parallel. The results showed a significant enrichment in *Streptococcus* in aged group compared to younger group (**Supplementary Figure 2**). However, there was no correlation between *Streptococcus and Candida* in any of the subgroups (**Supplementary Figure 3**). Interestingly, while there was a significant positive correlation between *Prevotella* and *Candida* in aged healthy control subgroup (**Supplementary Figure 4**), other potential pathobionts such as *Fusobacterium* did not show any correlation with *Candida* abundance (**Supplementary Figure 5**). Aging (1, 2, 19), HIV infection (28–30, 33–35) and microbiome changes are associated with changes in the mucosal immune cells (17, 18, 36), including CD4^+^CD25^+^FOXP3^+^T_regs_. Therefore, using flow cytometry, we assessed the proportions of oral gingival T_regs_ in young and old individuals, in healthy and HIV+ subgroups. It is well-established that HIV-positive individuals exhibit elevated levels of oral mucosal T_regs_ (2, 19, 29, 37). It further revealed that T_reg_ proportions are elevated both in aged healthy group and young HIV+ group, when compared to healthy young controls, dissociated from *Candida* abundance data (**Figure 3C**). These results showing increased *Candida* abundance in older subgroups of both healthy control and HIV^+^ groups, suggested an aging-dependent influence on *Candida* colonization irrespective of immune health or presence of chronic infection. However, chronic infection by itself was not associated with changes in *Candida* colonization. Thus, our data provide insights into the immune regulatory environment of the oral cavity, highlighting potential differences in age-related and infection-related variations in T_reg_ levels, which may be impacted by fungal colonization patterns.

Fungal components, particularly opportunistic commensals like *Candida albicans*, are capable of becoming pathogenic when host immunity is compromised, releasing pro-inflammatory mediators that contribute to disease progression. The cell wall components of fungi, such as β-glucans and mannans, act as PAMPs that trigger innate immune pathways via TLR-2 and dectin-1 receptors, leading to cytokine release and T-cell polarization (38). Chronic activation of these pathways by persistent fungal stimuli may drive immune imbalance and contribute to the sustained inflammatory state of inflammaging. Since TLR-2 signaling and cytokines can induce the proliferation of T_regs_ (16, 39–42), we examined the levels of specific inflammatory and dysbiosis markers, including soluble TLR2 (sTLR2), IL-6, and soluble CD14 (sCD14), in saliva.

**Figure 4:**
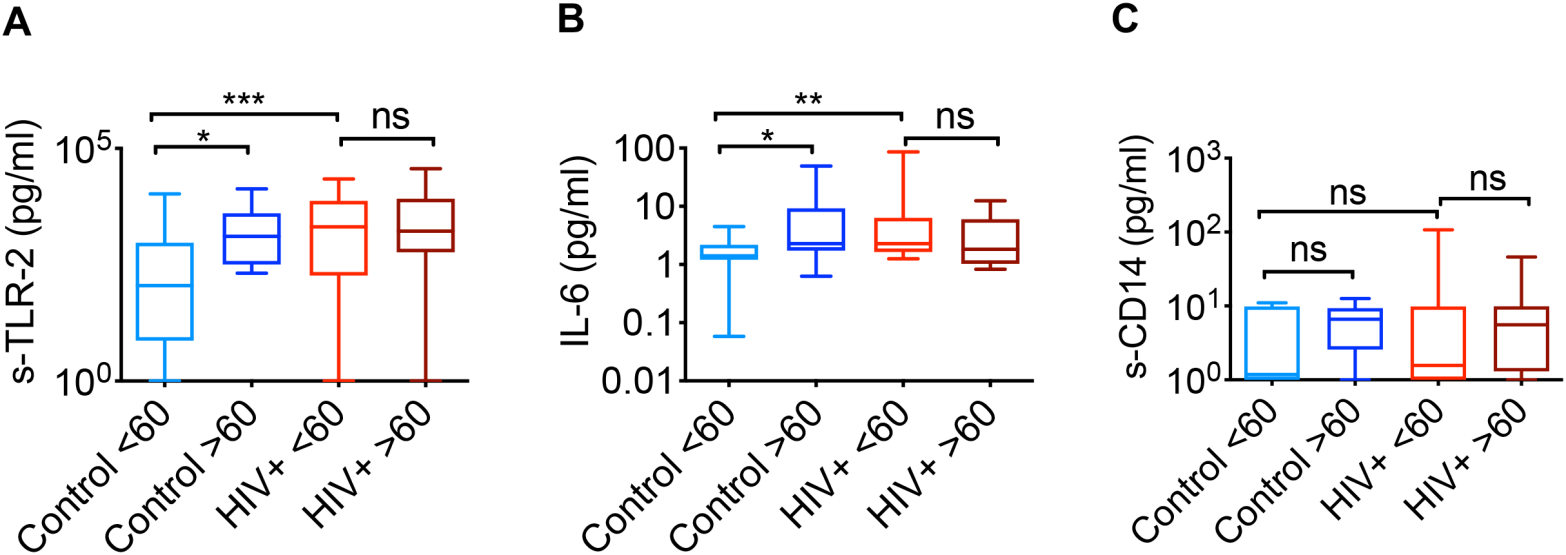
Salivary soluble TLR-2 and IL-6 levels increase with aging and in HIV+ individuals when compared to young control group. Soluble CD14 levels are comparable between the subgroups. **(A)** ELISA quantification of salivary sTLR-2 **(A)**, IL-6 **(B)**, and soluble CD14 **(C)** across subgroups stratified by age and HIV status. (Control, n=32; HIV+, n=46; *P <0.05 **P <0.005 ***P <0.0005; n.s, non-significant; two-tailed; Mann-Whitney test).

We focused on the cytokine IL-6, because IL-6-driven expansion of dysfunctional T_reg_ cells is physiologically relevant for oral inflammation during *Candida* infection in aged mice (19). We utilized ELISA to quantify these markers across different subgroups, stratified by age and HIV status. The quantification of salivary sTLR2 and IL-6 revealed that both aging and HIV infection are associated with elevated levels of these markers (**Figures 4A, B**). sTLR2, a component of the innate immune response, being upregulated, potentially reflects heightened TLR-2 signaling-mediated immune activation or inflammation in oral mucosa. IL-6, a key mediator of inflammation, being elevated in these populations, further supports the link between chronic inflammation and both aging and HIV infection. This coincided with increase in T_regs_ in these groups. In contrast, the quantification of sCD14, another marker of immune activation, did not show changes with either aging or HIV status (**Figure 4C**). This suggests that sCD14 an indicator of microbial translocation, frequently used in studies of gut barrier dysfunction may not change with aging in oral mucosa. Overall, these results highlight the similarities between aging and HIV infection with respect to specific inflammatory markers in saliva and increase in oral mucosal T_regs_, but not in *Candida* abundance. Because the levels of T_regs_, IL-6 and sTLR-2 were already elevated in young HIV+ subgroup compared to healthy counterparts, aging did not further increase these components in HIV+ groups.

### Direct correlation between *Candida* abundance in saliva and T_regs_ and CD4 hyperactivation in oral gingival mucosa

**Figure 5:**
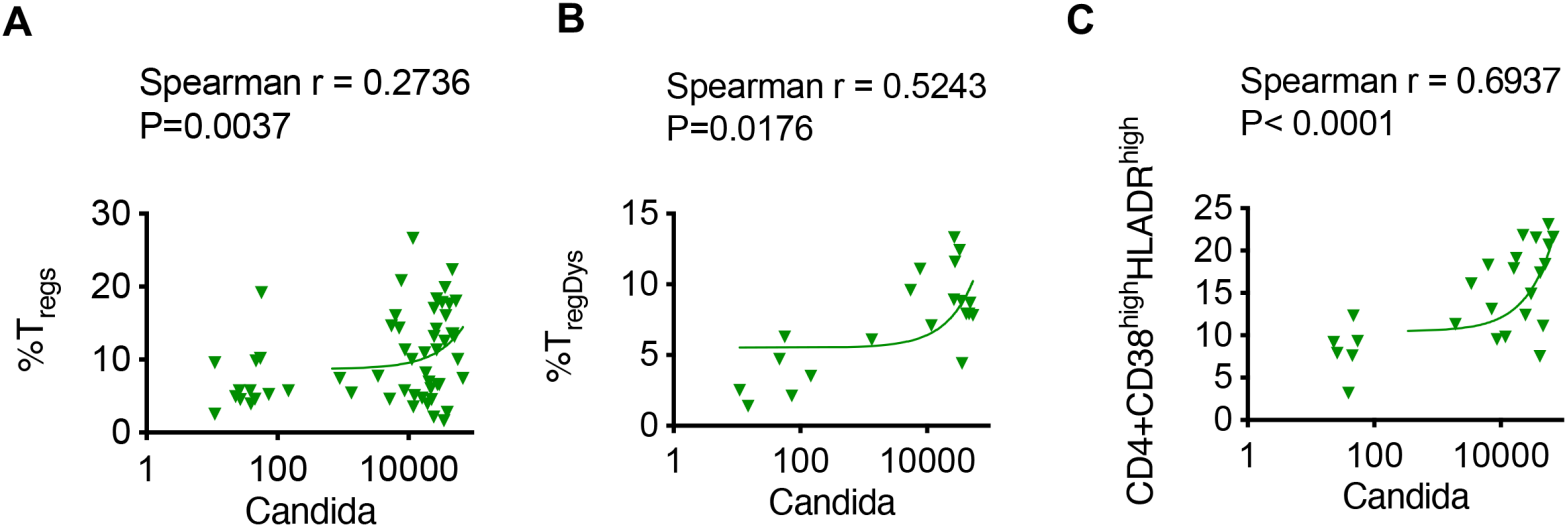
*Candida* abundance in saliva positively correlates with gingival T_regs_ T_regDys_ and CD4 hyperactivation in oral mucosa. Salivary ITS sequence mycobiome in conjunction with flow cytometry analysis of gingival immune cells were performed. Correlation between *Candida* and **(A)** gingival CD4^+^FOXP3^+^T_regs_; **(B)** CD4^+^FOXP3^+^IFN-γ^+^PD*-*1^+^(T_regDys_), and **(C)** CD4^+^CD38^+^ HLADR^high^ hyperactivated CD4^+^ cells.

Based on our previous data that have demonstrated *Candida*’s role in T_reg_ dysfunction in mice and humans (18, 19), we next explored the relationship between *Candida* abundance and T_regs_ in the oral gingival mucosa. We found a moderate positive correlation between the abundance of *Candida* in saliva and the levels of CD4^+^CD25^+^FOXP3^+^T_regs_ in the gingiva (**Figure 5A**). This suggests that as *Candida* levels increase, there is a concurrent rise in the population of T_regs_, which are crucial for maintaining immune tolerance and preventing excessive inflammatory responses. We have previously described FOXP3^+^ cells expressing both IFN-γ and PD-1 as functionally non-suppressive., i.e., dysfunctional (1, 2, 19). These T_regDys_ cells are indicative of impaired regulatory function and are often associated with chronic inflammation and cancer (28–30, 43). We found a stronger positive correlation between *Candida* abundance and the presence of dysfunctional T_regs_, characterized by the expression of IFN-γ and PD-1 markers in CD4^+^CD25^+^FOXP3^+^ cells (**Figure 5B**). The positive correlation suggests that higher *Candida* levels may contribute to, or result from, an increase in these dysfunctional cells and impaired oral immune regulation during aging. CD38 and HLADR have been previously described as markers of hyperactivation in conventional CD4 cells (44). Our results here showed a correlation between *Candida* abundance and CD4 hyperactivation in the oral gingival mucosa, further validating the role of functional impairment of T_regs_ in leading to overactivated CD4+ T cells and further contributing to inflammatory milieu (**Figure 5C**).

### *Candida* spp induces an increase in the percentage of FOXP3^+^ cells

**Figure 6:**
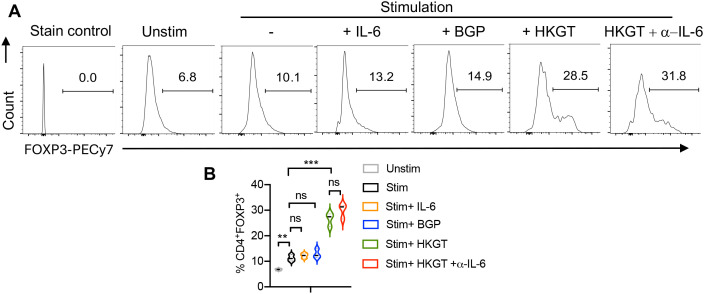
*Candida albicans* heat-killed germ tube (HKGT) but not beta glucan peptide (BGP) induces FOXP3+ cells in oral lymphoid T cell cultures. **(A)** Purified CD4^+^CD25^neg^ naïve cells from tonsils (~92% purity) were TCR-stimulated under induced-T_reg_ polarization conditions with or without IL-6, BGP, HKGT or HKGT+α-IL-6. Flow cytometric assessment of FOXP3 expression was performed on day 4 after stimulation.**(A)** Representative flow cytometric data (gated on CD4+ cells; top) and **(B)** statistical analyses from three independent tonsil donors (bottom) are shown. FOXP3 FMO control is shown as the staining control. Mean values +/- SEM are shown in statistical analysis; ***P<0.0005; **P<0.005. Two-tailed; Unpaired *t* test.

We next addressed the direct role of *Candida* spp in modulating T_reg_ induction and differentiation. We investigated the ability of different stimuli to induce FOXP3^+^ T_regs_ in T cell receptor (TCR)- activated human oral lymphoid organoid cultures. We purified CD4(+) CD25^neg^ naïve T cells and activated them in induced T_reg_ (iT_reg_) conditions in the presence of heat-killed *Candida albicans* Germ Tube (*Candida* spp; HKGT; a TLR-2 ligand), beta glucan peptide (BGP; a dectin-1 ligand), IL-6 and neutralizing IL-6 antibody. HKGT significantly induced the expression of FOXP3^+^ cells in the CD4^+^ T cell population (**Figures 6A, B**). Flow cytometric analysis was conducted on day 4 post-stimulation to assess FOXP3 expression. The data suggested that HKGT can promote the differentiation of naïve T cells into regulatory T cells, characterized by FOXP3 expression. In contrast, BGP did not induce a similar increase in FOXP3^+^cells, indicating that this fungal pathogen-associated molecular component does not have the same capacity to promote T_reg_ differentiation. Neither the inclusion of recombinant IL-6 nor the IL-6 blocking antibody with HKGT significantly altered the induction of FOXP3^+^ cells. This suggests that the effect of HKGT on T_reg_ differentiation is not mediated through IL-6 signaling. Overall, these results highlight the unique ability of *Candida* spp to promote the differentiation of FOXP3^+^ T_regs_ from naïve CD4^+^ T cells in the oral lymphoid environment, independent of IL-6 signaling. However, this finding underscores the potential role of changes in *Candida* colonization as a modulator of immune tolerance in the oral cavity.

### *Candida* spp induces the proliferation of CD4^+^FOXP3^+^ PD-1^+^IFN-γ^+^ dysfunctional cells in IL-6 dependent manner

**Figure 7:**
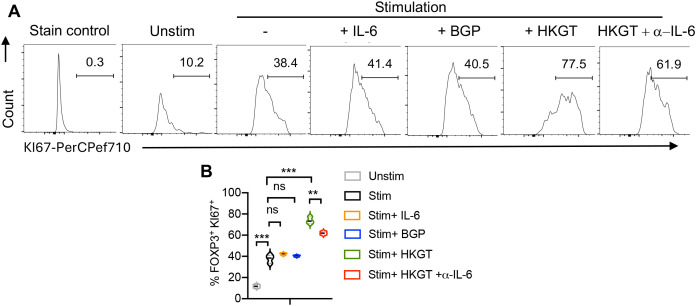
*Candida albicans* HKGT but not BGP increases proliferation in FOXP3+ cells in oral lymphoid T cell cultures. Purified CD4^+^CD25^neg^naïve cells from tonsils were TCR-stimulated under iT_reg_ polarization conditions with or without IL-6, BGP, HKGT or HKGT+α-IL-6. Flow cytometric assessment of proliferation by measuring K167 expression on day 4 after stimulation. Representative flow cytometric data (gated on CD4+FOXP3+ cells; **A**) and statistical analyses from three independent tonsil donors: **B** are shown. K167 FMO control is shown as the staining control. Mean values +/- SEM are shown in statistical analysis; ***P< 0.0005; **P<0.005. Two-tailed; Unpaired t test.

**Figure 8:**
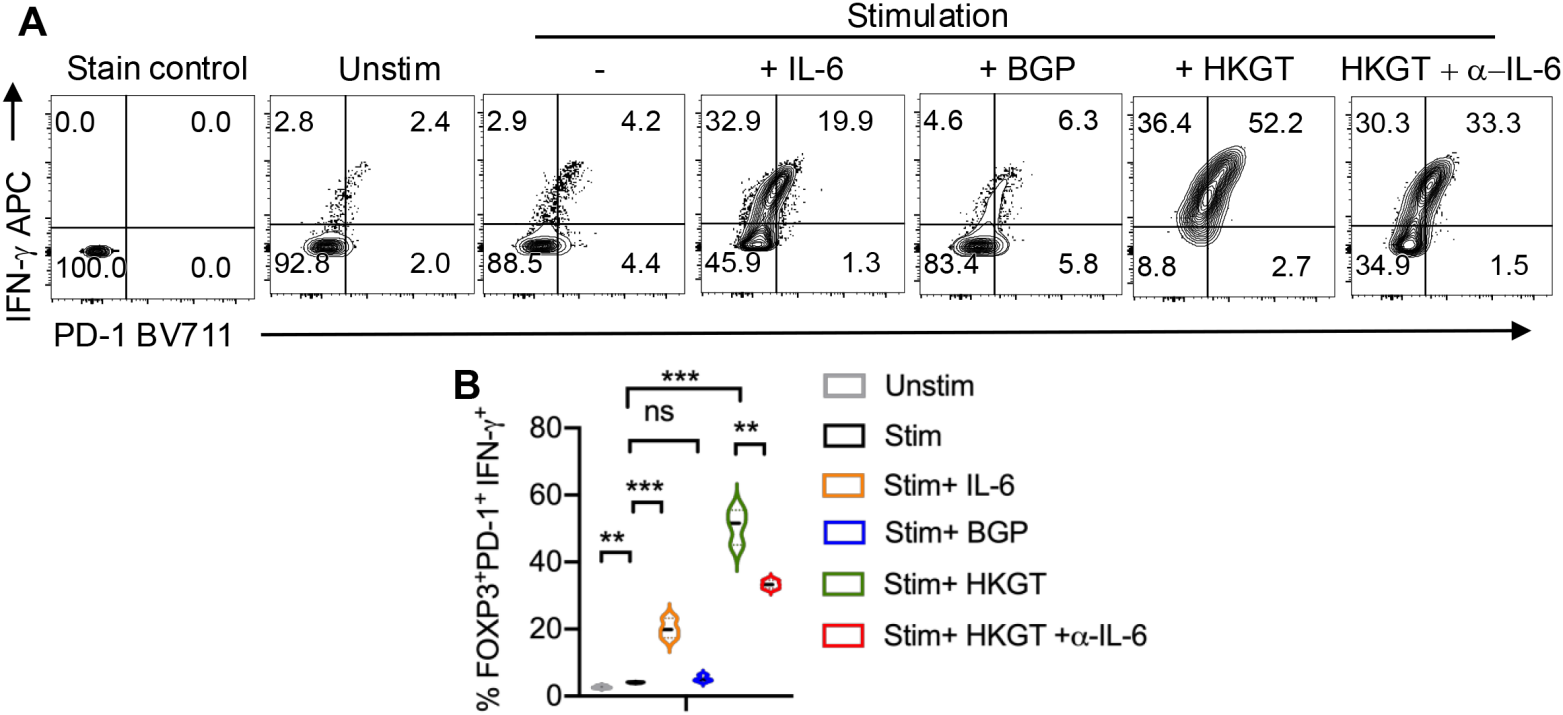
*Candida albicans* HKGT induces the proliferation of CD4^+^FOXP3^+^PD-1^+^IFN-γ^+^ dysfunctional cells in IL-6 dependent manner in oral lymphoid T cell cultures. **(A)** Purified CD4^+^CD25^neg^naïve cells from tonsils (~92% purity) were TCR-stimulated under iTreg polarization conditions with or without IL-6, BGP, HKGT or HKGT+α-IL-6. Flow cytometric assessment of PD-1 and IFN-γ expression on day 6 after stimulation. **(A)** Representative flow cytometric data (gated on CD4+FOXP3+ cells; top) and **(B)** statistical analyses from three independent tonsil donors (bottom) are shown. FMO control is shown as the staining control. Mean values +/- SEM are shown in statistical analysis; ***P< 0.0005;**P< 0.005. Two-tailed; Unpaired t test.

We then assessed cell proliferation by measuring the expression of KI67, a well-known marker of cell proliferation, and found that HKGT significantly increased the proliferation of FOXP3^+^ cells, indicating that HKGT not only promotes the differentiation of these regulatory T cells but also enhances their proliferative capacity. This suggests a potential role for HKGT in expanding the population of regulatory T cells within the oral lymphoid environment (**Figures 7A, B**). In contrast, BGP did not induce a similar increase in proliferation, highlighting a distinct difference in the capacity of these fungal components to influence T cell dynamics. The presence of IL-6 alone did not significantly alter the proliferation of FOXP3^+^ cells. However, in the presence of HKGT, addition of IL-6 blocking antibody reduced the proliferative effect of HKGT suggesting that proliferation of FOXP3^+^ cells is dependent on IL-6 signaling. We then focused on CD4^+^FOXP3^+^ cells to assess the expression of PD-1 and IFN-γ markers indicative of dysfunctional T_reg_ cells. We and others have previously described IFN-γ and PD-1 expressing FOXP3+ as functionally non-suppressive (29, 30).We found that *Candida* spp induced a significant proliferation of these dysfunctional FOXP3^+^iT_reg_ cells, characterized by the expression of PD-1 and IFN-γ (**Figures 8A, B**).

**Figure 9:**
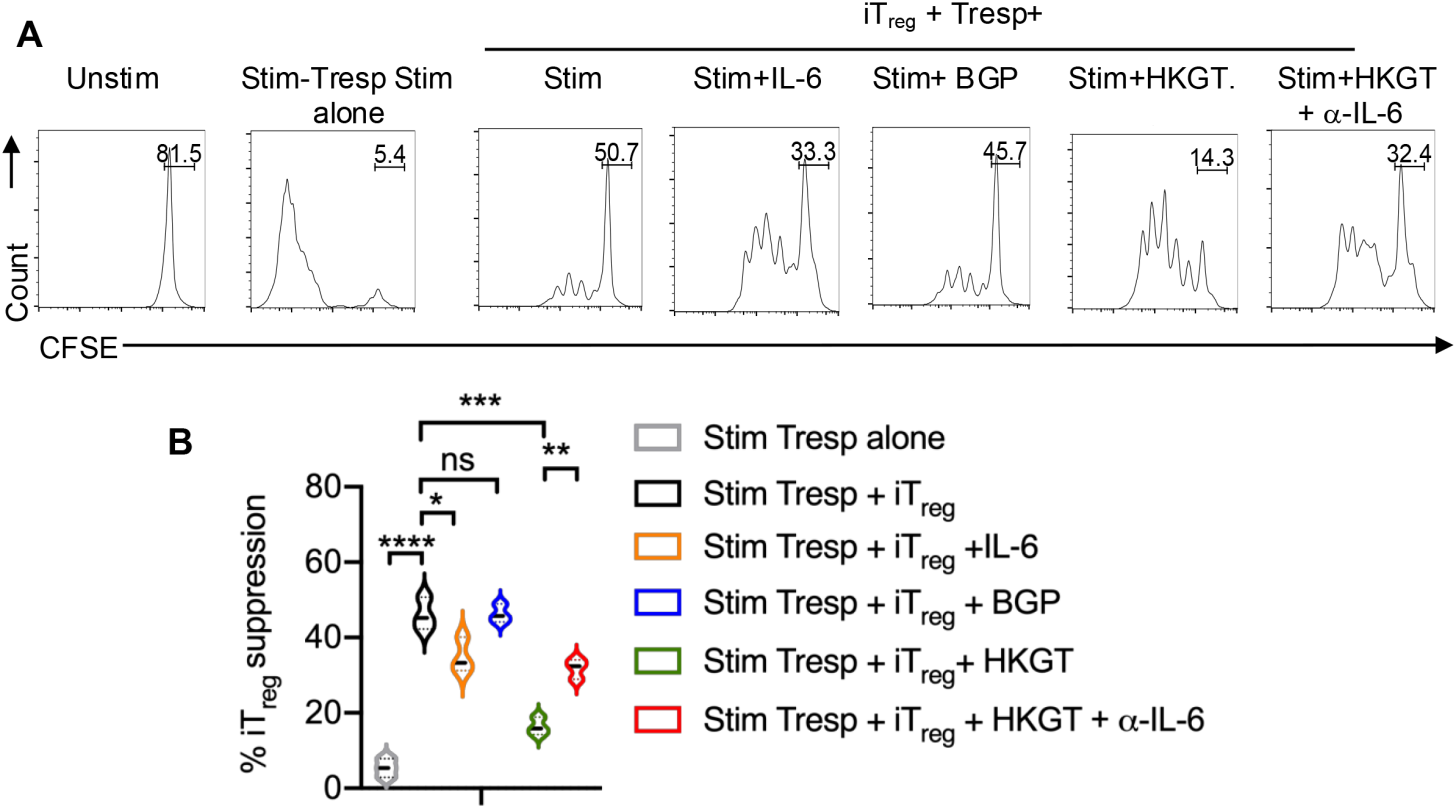
*Candida albicans* HKGT induces non-suppressive FOXP3+ cells (dysfunctional) in IL-6 dependent manner. Purified CD4^+^CD25^neg^naïve cells from tonsils (~92% purity) were TCR-stimulated under iT_reg_ polarization conditions with or without IL-6, BGP, HKGT or HKGT+α-IL-6 for 6 days after stimulation. CD4^+^CD25^+^ cells were purified from these iT_reg_ cultures and replated in co-cultures along with fresh CFSE-labeled naïve responder (Tresp) cells in a suppression assay. Proliferation suppression was measured in Tresp cells, 5 days after the co-culture. Tresp cells were stimulated (Stim) alone as a control (2^nd^ column). **(A)** Representative flow cytometric data (gated on CFSE labeled Tresp cells, and **(B)** statistical analyses of % suppression in Tresp by iT_regs_ from three experiments are shown. Mean values +/- SEM are shown in statistical analysis; * P<0.05, *** <0.0005, ****P<0.0005. Two-tailed; Unpaired t test.

To determine their suppressive function, we also stimulated the CD4^+^CD25^+^ cells that were purified from these iT_reg_ cultures and replated in co-cultures along with fresh CFSE-labeled naïve responder (Tresp) cells in a suppression assay. Proliferation suppression was measured in Tresp cells, 5 days after the co-culture. As a control, Tresp cells were stimulated alone. The results showed that iT_regs_ from *Candida* spp cultures had reduced suppressive activity as determined by their ability to suppress proliferation of Tresp cells (**Figures 9A, B**). We also observed that neither BGP nor IL-6 alone was sufficient to induce a similar proliferation of dysfunctional T_regs_.

## Discussion

Aging is associated with inflammaging, a state of chronic, low-grade systemic and mucosal inflammation that develops in the absence of overt infection (1, 2, 19). A key unresolved question is whether fungal dysbiosis is a cause or a consequence of these aging-related inflammation. Our previous mouse data demonstrated that fungal burden is comparable between young and aged mice following *Candida* infection, suggesting that aging *per se* does not necessarily increase *Candida* burden during pathological infection (19). However, the impact of commensal *Candida* colonization in the context of an intact microbiome was not previously evaluated. Addressing this gap, our current work demonstrates that resident fungal dysbiosis, which includes *Candida* abundance contributes to inflammation, at least in part, by impairing T_reg_ function in the mucosa. Also, previous studies did not study age-related dysfunction comparing healthy aging and aging population with a chronic infection. Therefore, we characterized the oral mycobiome in aging groups in relation to immune health in healthy and HIV+ individuals with established oral inflammation. We have identified that older individuals in both groups show a higher prevalence of *Candida*, which is linked to changes in immune regulation as indicated by increased T_reg_ proportions as well as their dysfunction. With Shannon Index that measures both richness and evenness of species, our data also show significantly reduced diversity in aged individuals (**Figure 1C**). The decrease in fungal diversity aligns with the well-documented decrease in bacterial diversity in the elderly. Also, this finding suggests that in the context of the mycobiome, the qualitative shift towards a higher prevalence of opportunistic and potentially pro-inflammatory fungal genera (e.g., *Candida* and *Aspergillus*) might be more critical than the overall diversity metric (**Figures 2**, **3**). This data also imply that the specific identity and functional potential of the fungal species present, rather than merely their number, are paramount in influencing immune responses and contributing to age-related pathologies. Aligning with this notion, *Candida* levels were associated with increased T_reg_ and T_regDys_ populations, as well as heightened CD4+ T cell activation, which have previously been demonstrated in both aged mice and humans as well as chronic inflammation (1, 2, 19, 44). Microbial products and ligands including those from microbiome-derived agents play a direct and significant role in influencing the differentiation and function of T_regs_ (17, 18, 39). For instance, microbial metabolites such as short-chain fatty acids (SCFAs) have been shown to directly induce T_reg_ differentiation and enhance their immunomodulatory activity in the colon (45). Antibiotic-mediated depletion of resident bacteria diminishes the frequency of Foxp3^+^T_reg_ cells and T_reg17_ cells in the oral mucosa. This reduction in T_regs_ is accompanied by increased tissue pathology and a higher fungal burden during oral *Candida* infection, underscoring the vital role of commensal bacteria in controlling T_regs_ and Th17 cells, and in maintaining overall mucosal immune homeostasis (41, 42). However, the relationship between oral mycobiome and oral mucosal T_regs_ was never studied before. Our results here provide crucial insights into this subject, by showing that oral *Candida* abundance can cause elevated levels of dysfunctional FOXP3^+^ T_regs_ during aging (**Figures 3**-**5**). T_regs_ that can be induced in the periphery i.e., peripheral/induced T_regs_ (pT_regs_/iT_regs_) can acquire distinct repertoires shaped by environmental and tissue antigens, leading to tissue-specialized (and sometimes oligoclonal) expansions (46). Peripheral antigen exposure (microbiota, environmental antigens, tissue-restricted self-antigens) can drive tissue-resident pT_reg_ induction and local antigen-driven clonal expansion and transcriptional specialization. Single-cell paired TCR studies also show some tissue-restricted clonotypes (47). However, the TCR specificity of the dysfunctional T_regs_ we have uncovered in this study is unknown and requires future studies.

A critical observation in this study is that not only quantity of T_regs_ increases with age, but their quality or suppressive functionality appears to decline, likely contributing to inflammaging, a central hallmark of aging. Given their critical role in suppressing immune responses and maintaining mucosal immune homeostasis, age-related accrual of mucosal dysfunctional T_regs_ may also be a key mechanistic driver to inflammaging (48). Aligning with this notion, our results here showing a positive correlation between *Candida* abundance, dysfunctional T_regs_, and CD4 hyperactivation in the oral gingival mucosa, validate the role of functional impairment of T_regs_ in contributing to overactivated CD4+T cells and inflammatory milieu (**Figure 5**). CD4 hyperactivation reflects an overactive immune response, which can lead to tissue damage and inflammation. Therefore, an association with increased *Candida* levels implies a potential feedback loop where increased colonization exacerbates mucosal immune activation, further impacting mucosal health. Thus, the research establishes a clear link between age-related oral dysbiosis and chronic inflammation. T_regs_ function through multiple mechanisms in mucosa and predominantly function through IL-2 consumption effector cells and require TGF-β for their survival during oral *Candida* infection (16, 19, 27, 41, 42, 49). During *Candida albicans* infection, T_regs_ have been reported to have dual protective functions through enhancing Th17 immunity and controlling mucosal immunopathology (50). However, dysfunction in T_regs_ renders their immunomodulatory functions ineffective. Our study here clearly delineates a mechanism by which specific microbial ligands and cytokines such as soluble TLR-2 and IL-6 may cause T_reg_ dysfunction, causing a qualitative shift in the immune response towards a more pro-inflammatory state in aging.

Taken together, our previous studies (28, 29) and the current study suggest that aging and chronic HIV infection can result in similar functional inflammatory effects via T_reg_ dysregulation, but through distinct mechanisms within the mucosal environment. Mechanistically, both the proliferation of dysfunctional T_regs_ and lack of suppressive activity were found to be dependent on *Candida* spp stimulation and IL-6, as evidenced by the reduced proliferation (**Figure 7B**), lower frequency of PD-1 and IFN-γ expressing FOXP3^+^ cells (**Figure 8B**), and enhanced suppression (**Figure 9B**), when IL-6 was blocked. This indicates that IL-6 plays a crucial role in the differentiation and expansion of subset of T_regs_ including dysfunctional T_regs_ in response to persistent *Candida* spp. Absence of any effect by BGP effectively rules out the role of dectin-1 signaling or cytokine alone in T_reg_ dysfunction. Importantly, our data emphasize the specific impact of the combined actions of *Candida* spp and endogenous IL-6 in facilitating T_regDys_ proliferation. Thus our work corroborates our previous studies showing that elevated *Candida* are associated with gut inflammation (22) and *Candida*-driven oral immunopathology during bacterial dysbiosis and aging (18, 19).

Limitations of the study: Aging arises from a multifaceted interplay of genetic, environmental, and stochastic factors. Our study focused on just two dimensions, the oral mucosal microbiome and the immune system, and are thus not comprehensive. Our previous publication demonstrating that *Candida* infection causes T_reg_ dysfunction and gut inflammation in aged mice (18, 19), and the present work showing that HKGT directly increases T_reg_ dysfunction in the context of IL-6 led us to interpret that aging increases *Candida* burden, which subsequently causes T_reg_ dysfunction in the oral mucosa. However, the findings should be interpreted with caution and other confounding variables should be considered in the future studies. Also, while the potential synergistic or antagonistic interactions among other bacteria and fungi with T_reg_ dysfunction are extremely interesting aspects of future studies, this study focused on the role of *Candida*, based on our previous data that have demonstrated *Candida*’s role in T_reg_ dysfunction in mice and humans (18, 19). Moreover, our preliminary correlative analysis between other prominent fungi and T_regs_ did not yield significant results in this study (data not shown). We plan to perform similar mycobiome analysis in the next cohort. Combining the future data and increasing the overall “n” may increase the statistical power for comparisons. Including potential broader demographic profile (e.g., geographic, socioeconomic) would enhance the generalizability of the future findings. HIV+ cohort in this introduces confounding variables, such as antiretroviral therapy and duration of infection, that are difficult to fully control, making direct comparisons with immunosenescence in healthy aging complex. As shown in **Figures 1**, **3A**, approximately 50% of younger individuals exhibit high *Candida* levels. However, the significant increase in *Candida* abundance observed in the older group remains consistent regardless of the number of samples analyzed. **Figure 3B** demonstrates that, even when analyzing fewer samples after separating HIV-negative (healthy) and HIV-positive individuals, there remains a significant increase in *Candida* abundance in the older group (compare the first two violin plots in **Figure 3B**). Additionally, these data indicate that many of the younger individuals with high *Candida* levels are found within the HIV-positive group (see the third violin plot in **Figure 3B**), suggesting that most younger healthy individuals have low *Candida* levels. In contrast, among healthy individuals, 100% of the older group are *Candida* high (compare the first two violin plots in **Figure 3B**). Thus, our comparison analysis, separating the healthy and HIV+ cohort and showing a comparison between A1 and A2 non-HIV groups shows a significant enrichment of *Candida* and inflammatory parameters even during healthy aging (**Figures 3B, C**, **4A-C**). Increased *Candida* abundance in older healthy group may predict features of inflammaging. Future multi-omics approaches that incorporate key hallmarks of aging, including inflammation, senescence, and autophagy, will be essential for advancing mechanistic insight. Additionally, we did not investigate the influence of metabolic interventions or pharmacological strategies targeting the features identified here, which may represent promising avenues to support healthy aging. However, by addressing complex interconnections between mycobiome and key immunoregulatory cells in mucosa, our research offers fresh insights that may help address aging-related diseases and bolster immune resilience throughout life.

## Materials and methods

### Subject recruitment and sampling of human gingival biopsies and saliva

Human gingival biopsies and saliva samples were collected from participants, including healthy controls (healthy Control; n=32) and individuals living with HIV (n=46), following a protocol approved by the Institutional Review Board at University Hospitals Cleveland Medical Center. Informed consent was obtained from all participants prior to sample collection (28, 29). The characteristics of the participants enrolled for gingival biopsy and saliva collection are detailed in **Supplementary Table 1**. These participants are from the same cohort as those in our previous studies (28, 29). Healthy control subjects were 18 years of age or older and in good general health. Exclusion criteria included the presence of oral inflammatory lesions (such as gingivitis and periodontitis), a diagnosis of oral cancer, soft tissue lesions, and tobacco use within the past month. Tobacco abstinence was verified by measuring salivary cotinine using ELISA. HIV+ participants were also ≥18 years old, had confirmed HIV infection, and had been on cART for at least one year. More than 35% reported prior candidiasis and 75% reported previous or current soft-tissue lesions, gingivitis, or periodontitis. CD4+ T-cell counts ranged from approximately 350–700/μl in both healthy controls and HIV+ participants. Single-cell suspensions of gingival tissues were prepared following Collagenase 1A digestion. As indicated in the results, only a subset of gingival and saliva samples was available for the paired correlation analysis described in this study. For human tonsil organoid cultures (HTOC), discarded palatine tonsils were obtained from tonsillectomies at University Hospitals Cleveland Medical Center through the Histology Tissue Procurement Facility, under a separate IRB-exempt protocol. Gingival biopsies were processed fresh for flow cytometry, while tonsil cells, either fresh or cryopreserved, were analyzed using flow cytometry or utilized to establish HTOC cultures. Single-cell suspensions of gingival and tonsil tissues were prepared by digesting with Collagenase 1A (0.5 mg/mL0.5mg/mL; Sigma C9891), followed by centrifugation with Ficoll-Paque PLUS (GE17-1440-02; Millipore Sigma) at 900g and subsequent PBS washes. Mouse experiments were conducted at Case Western Reserve University (CWRU) under approval from the CWRU Institutional Animal Care and Use Committee, adhering to all guidelines and regulations.

### Microbiome sequencing

Sample Collection and DNA Extraction: A total of 66 human saliva samples were processed for 16S rRNA and ITS1 sequencing as described previously (21, 51, 52). Microbial DNA was extracted from 50 µL of saliva per sample using the QIAamp PowerFecal Pro DNA Kit (Qiagen), following the manufacturer’s protocol. Positive and negative controls included the ZymoBIOMICS™ Microbial Community Standard (Zymo Research Corporation) and UltraPure™ DNase/RNase-Free Distilled Water (Invitrogen), respectively, which were processed alongside the samples. The PCR reaction was conducted in a reaction well plate with the following components: 5 µL template DNA, 2.5 µL Gold Buffer (10X), 2.5 µL MgCl_2_ (25 mM), 0.5 µL dNTP (10 mM), 1 µL each of the 16S or ITS1A primers (10 µM), 0.25 µL AmpliTaq Gold DNA Polymerase (5 U/µL), and 12.25 µL UltraPure DNase/RNase-free distilled water (Invitrogen). Fungal ITS1 amplicons were amplified using primers specific to recombinant DNA target regions: ITS1 forward (5′-CTTGGTCATTTAGAGGAAGTAA) and ITS2 reverse (5′ GCTGCGTTCTTCATCGATGC). Bacterial 16S rRNA V4 region was amplified with primers (53): forward 5’- GTGCCAGCMGCCGCGGTAA-3’ and reverse 5’- GGACTACHVGGGTWTCTAAT-3’. The PCR amplification conditions were set to 95 °C for 3 minutes, followed by 28 cycles of 95 °C for 30 seconds, 55 °C for 30 seconds, and 72 °C for 30 seconds, concluding with 72 °C for 5 minutes. Amplicons were purified using Ampure XP Beads and verified with the TapeStation 4150 (Agilent). DNA quantification was performed using Quant-iT™ dsDNA Assay Kits HS (Invitrogen) and normalized to 4 nM. The pooled library was diluted to 8 pM with a 10% spike-in of 12.5 pM PhiX before sequencing on the Illumina MiSeq, employing 500 cycles v2 PE reagents to produce 2 x 250 bp paired-end reads (Illumina). Data Processing: Sequence reads underwent quality control using the DADA2 pipeline (version 1.26). Paired-end reads for each sample were assembled into contigs, and primer sequences were trimmed. Sequences were classified using the Ribosomal Database Project (RDP) taxonomy database (version 16) or the UNITE fungal database. Taxa detected at higher average levels in the negative water controls than in the samples were identified as contaminants and removed. Operational Taxonomic Units (OTUs) were assigned based on their most specific taxa hits prior to downstream analysis. OTUs were labeled as “undistinguishable” if the database could not classify the sequence beyond domain or class. Low abundance taxa, representing less than 0.025% of total species detected across all samples, were categorized as “other.” Quality Control: Sequence quality control and denoising were performed using QIIME 2 (v2024.5), an open-source bioinformatics pipeline for microbiome analysis. Low-quality bases (quality score< 30) at sequence ends were trimmed, and sequences shorter than 100 nucleotides were excluded. The DADA2 plugin filtered and corrected sequencing errors, inferred amplicon sequence variants (ASVs), and removed chimeric sequences. The first 15 bases of both forward and reverse reads were trimmed to eliminate primers and low-quality regions, while forward and reverse reads were truncated at 240 bp and 220 bp, respectively, to retain high-quality regions. This approach ensured the removal of sequencing errors while preserving high-resolution ASVs for downstream taxonomic and diversity analyses. Taxonomy Assignment: Fungal ITS1 sequences were subsequently trimmed with ITSxpress (v1.7.4) within the QIIME 2 package (v2019.7). To minimize sequencing noise and consolidate similar sequences, *de novo* OTU clustering was performed using VSEARCH within QIIME 2. Sequences were clustered at 99% sequence identity, grouping highly similar sequences into the same OTU. For bacterial microbiome analysis, filtered ASVs were taxonomically classified using the QIIME 2 feature-classifier plugin, which applies a naïve Bayes classifier trained on the SILVA reference database (54, 55). Taxonomic assignment was conducted using a pre-trained naïve Bayes classifier based on SILVA, employing a 99% sequence identity threshold. The resulting OTU tables and taxonomy assignments were subsequently integrated into two phyloseq objects in.RData format for downstream statistical and visualization analyses (56). The phyloseq framework streamlined microbiome data management, enabling efficient implementation of alpha diversity, beta diversity, and differential abundance analyses. Alpha Diversity Analysis: Alpha diversity was evaluated using two metrics: the Shannon index and the Chao1 richness estimator. The Shannon index considers both species richness and evenness, providing a measure of diversity within each sample. In contrast, the Chao1 index estimates species richness with a focus on accounting for rare taxa. These metrics were calculated for each sample using the vegan package in R (v4.4.1). Differences in alpha diversity between the two groups were assessed, with statistical significance evaluated using the Mann-Whitney U test, a non-parametric method suitable for comparing distributions between two independent groups. Multiple testing corrections were applied where necessary to control for false discovery rates. Beta Diversity Analysis: Beta diversity was assessed to compare microbial community composition across samples using the weighted UniFrac distance metric (57), a phylogenetic-based measure that accounts for both taxonomic relatedness and relative abundance. Weighted UniFrac distances were calculated using the GUniFrac package in R. Differences in microbial community composition between the groups were evaluated through permutational multivariate analysis of variance (PERMANOVA) on pairwise weighted UniFrac distances, utilizing the GUniFrac package function. To visualize beta diversity patterns, principal coordinate analysis (PCoA) was performed on the weighted UniFrac distance matrix using the cmd scale function from the R base package. The first two principal coordinate axes were plotted to capture the primary sources of variation in microbial community structure across samples. Differential Abundance Analysis: To identify microbial taxa that significantly differed between the two groups, differential abundance analysis was conducted using the Linear Discriminant Analysis Effect Size (LEfSe) method (58). LEfSe is a statistical method designed to identify biologically meaningful differences in microbiome composition by combining non-parametric statistical tests with Linear Discriminant Analysis (LDA). LEfSe analysis was conducted using the microbiome Marker package in R. For microbiome data, Wilcoxon rank-sum (for two-group comparisons) and Kruskal-Wallis (for multi-group comparisons) tests were employed with a significance threshold of p<0.05. An LDA score cutoff of 2.0 was set to select strongly discriminative microbial features. To control for multiple testing, p-values were adjusted using the False Discovery Rate (FDR) correction. To visualize the hierarchical structure of microbial biomarkers identified by LEfSe, a taxonomy tree was constructed using the BAZE package. This representation aids in interpreting the taxonomic relationships among differentially abundant microbial taxa. For key microbial markers identified through LEfSe analysis, we further examined their association with the Control and PLWH groups using the Wilcoxon rank-sum test. To ensure robust statistical inference, p-values were adjusted using both FDR correction and Bonferroni adjustment, minimizing the risk of false-positive findings. This stringent approach enhances the reliability of conclusions regarding the significance of microbial differences between the groups.

### iT_reg_ suppression assay *in vitro*

For the suppression assay, 5× 10^4^ CD4^+^CD25^+^ cells purified from iT_reg_ cultures were co-cultured with purified 5× 10^4^ CD4^+^CD25^−^ responder T cells (Tresp) cells in U-bottom 96-well plates. iT_regs_ were labelled with CellTrace Violet (Thermo Fisher Scientific) and Tresp cells were labeled with carboxyfluorescein succinimidyl ester (CFSE) (Thermo Fisher Scientific), fluorescent cell-tracking dyes before co-culturing in triplicate wells in the presence of soluble α-CD3(1 μg/ml) and α-CD28 (2 μg/ml) antibodies for 4 days. The proliferation of Tresp cells was measured by CFSE dilution.

### Antibodies and ELISA kits

Fluorochrome-conjugated antibodies were sourced from various suppliers as follows: CD3 (HIT3a), CD25 (M-A251), PD-1 (EH12.1), CD38 (HIT2) and HLA-DR (LN3) antibodies were purchased from BD Biosciences (PA, US). CD4 (OKT-4), FOXP3 (236A/E7), CD3 (HIT3a), and IFN-*γ*(4S-B3) antibodies were purchased from ThermoFisher Scientific (CA, US). TCR stimulating antibodies used in this study included CD3 (HIT3a) antibodies from BD Biosciences (PA, US) and CD28 (CD28.2) from Life Technologies Corporation (CA, US). Recombinant human TGF-β1 and IL-2 was procured from R&D Systems and BioBasic Inc. (NY, US) respectively. Soluble TLR-2, Soluble CD14, and IL-6 ELISA kits were from Boster Bio (Pleasanton, CA).

### Cell stimulation *in vitro*

HTOC cells were stimulated in U-bottom 96 well plates using 1 µg/ml of plate-bound α-CD3 and 2 µg/ml of α-CD28 antibodies with TGF-β1 (2 ng/ml), and IL-2 (36) in the presence of HKGT for 3–6 days, as indicated. CD4 naïve cell isolation kits were also used, and were purchased from Stem cell Technologies (Vancouver, Canada). Cells were cultured in complete RPMI-1640 (Hyclone) supplemented with 10% human serum, 100 U/ml penicillin, 100 µg/ml streptomycin, 2 mM glutamine, 10 mM HEPES, 1 mM sodium pyruvate and 50 µM β-mercaptoethanol. Heat killed *Candida albicans* germ tubes (HKGT) were generated in the laboratory by heat killing the germ tubes at 75 °C for 60 minutes. Germ tubes from *Candida* spp were prepared by growing blastospores (10*9/ml) in complete RPMI-10 at 37 °C with CO2 for 4–6 hours, or until the budding of germ-tubes.

### Intracellular staining of cytokines and flow cytometry

For single-cell flow cytometry staining, cells were cultured as described and then washed in PBS or PBS/BSA prior to surface staining with the appropriate antibodies. For Foxp3 staining, cells were fixed using the Foxp3 fix-perm set (eBioSciences/Thermo Fisher) following surface staining. Live-Dead viability staining was employed to exclude dead cells from the analyses. Appropriate controls, including unstained, isotype, secondary antibody, single stain, and FMO controls, were utilized. Before intracellular cytokine staining, cell cultures were re-stimulated with PMA (50 ng/mL) and Ionomycin (500 ng/mL) for 4 hours, with brefeldin-A (10 µg/mL) added during the last 2 hours. Data acquisition was performed using BD Fortessa cytometers, and the data were analyzed using FlowJo software versions 9.8 or 10.5.3.

### Statistical analyses

P-values were calculated using Prism 8 (GraphPad Software) to assess statistical significance, with a threshold of p<0.05 considered significant. For comparisons involving random distributions, the Mann-Whitney test was applied. One-way and two-way ANOVA analyses were used for multiple group comparisons, with Bonferroni t-tests serving as *post hoc* tests for multiple comparisons. To examine changes between groups, the unpaired two-sided Wilcoxon signed-rank test was utilized. For correlation analyses, Spearman correlation coefficients ( r) and simple linear regression (R^2^) values were calculated, with an alpha value of<0.05 deemed significant. All statistical analyses assumed random distribution and were conducted in Prism 6.1 or Prism 8 (GraphPad Software, Inc.).

## Data Availability

The 16SrRNA sequencing results generated in this study are deposited in Qiita at https://qiita.ucsd.edu/study/description/16115 and the study accession number ID is 16115.
