# Supplementary material for: Enrichment of Candida associated with dysbiosis contributes to mucosal CD4+FOXP3+ regulatory T cell accrual and their dysfunction in aging
Source: Front Immunol. 2026 Mar 19;17:1714595. doi: 10.3389/fimmu.2026.1714595 (PMC13043350; doi:10.3389/fimmu.2026.1714595)
Supplement: Supplementary file 1 [file DataSheet1.pdf]

Supplementary Table-1 Human participants enrolled in the study*

Group	HIV− (n = 32)	HIV+ ART (n = 46)
Age (years) median	49 +/- 9.9	54 +/- 18.5
Aged 60 and above	21.8%	22.2%
% females	59.3%	22.2%
Time under cART median	0	15 +/- 7.6 yrs
Viral load median (range) copies/ml	0	20 (0.8 - 272)
% prior oral Candidiasis positive	0	34.9%
% Periodontitis +	0	4.7%

*** Published- References 11, 28, and 29**

Data availability

The 16SrRNA sequencing results generated in this study are deposited in Qiita at https://qiita.ucsd.edu/study/description/16115 and the study accession number ID is 16115.

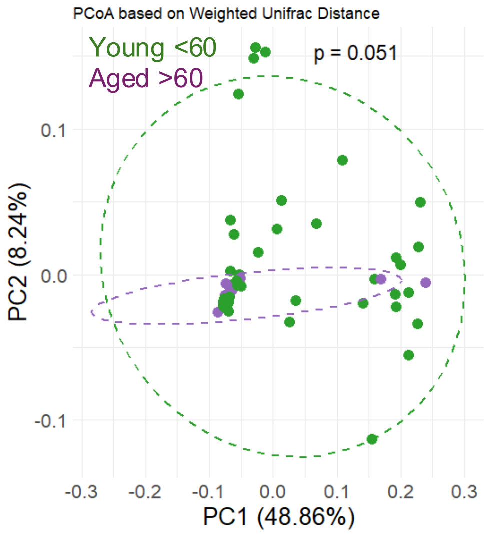

Supplementary Figure.1. Oral Mycobiome analysis. Principal coordinates analysis (PCoA) based on weighted UniFrac distance was used to assess age-related differences in oral fungal beta diversity. Each point represents an individual sample, with 95% confidence ellipses shown for the older (purple) and younger (green) groups. PC1 and PC2 explain 48.86% and 8.24% of the total variance, respectively. Although the PERMANOVA test was not satisfactorily statistically significant ($p = 0.051$), the broader dispersion observed in younger individuals may reflect greater inter-individual variability in their mycobiome composition.

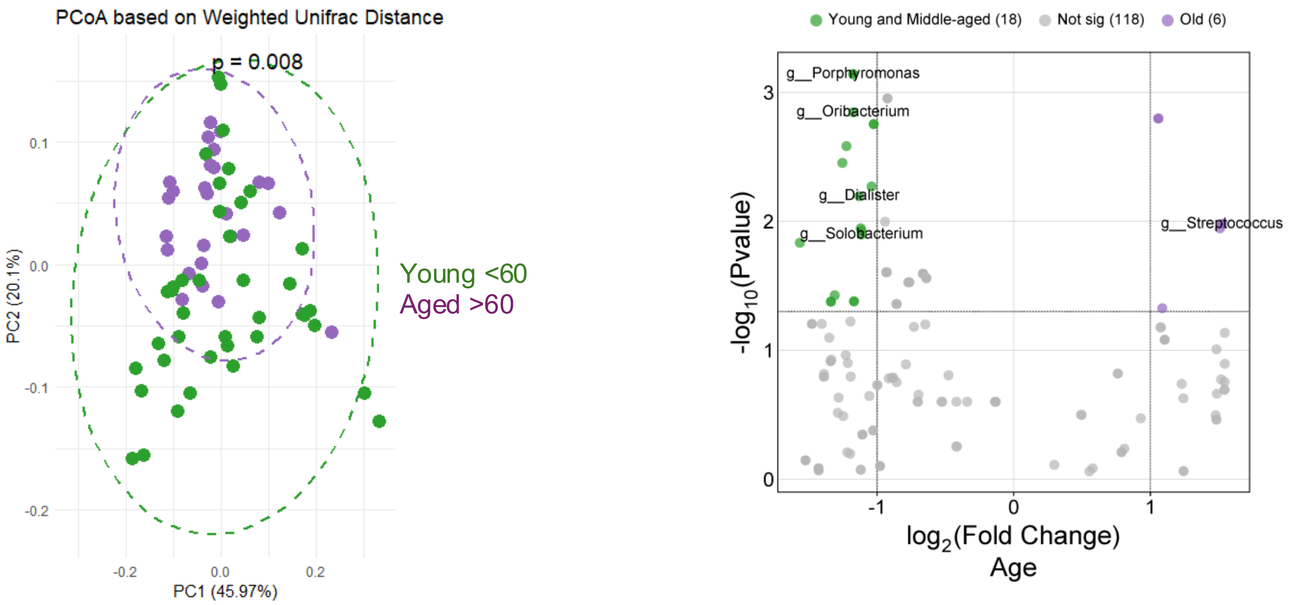

Supplementary Figure 2. Oral Microbiome analysis shows enrichment in Streptococcus in aged group. 16SrRNA gene sequencing was performed using the saliva DNA samples collected from the participants (n=66) and PLWH. PcoA analysis based on Bray-Curtis distance matrix (left) Volcano plot displaying differential abundance of bacterial taxa between young and old individuals. The x-axis indicates the $\log_2$ fold change (Young vs Aged), and the y-axis represents $-\log_{10}$ p-values (right). Statistically significant taxa enriched in the younger (green) and older (purple) groups are highlighted.

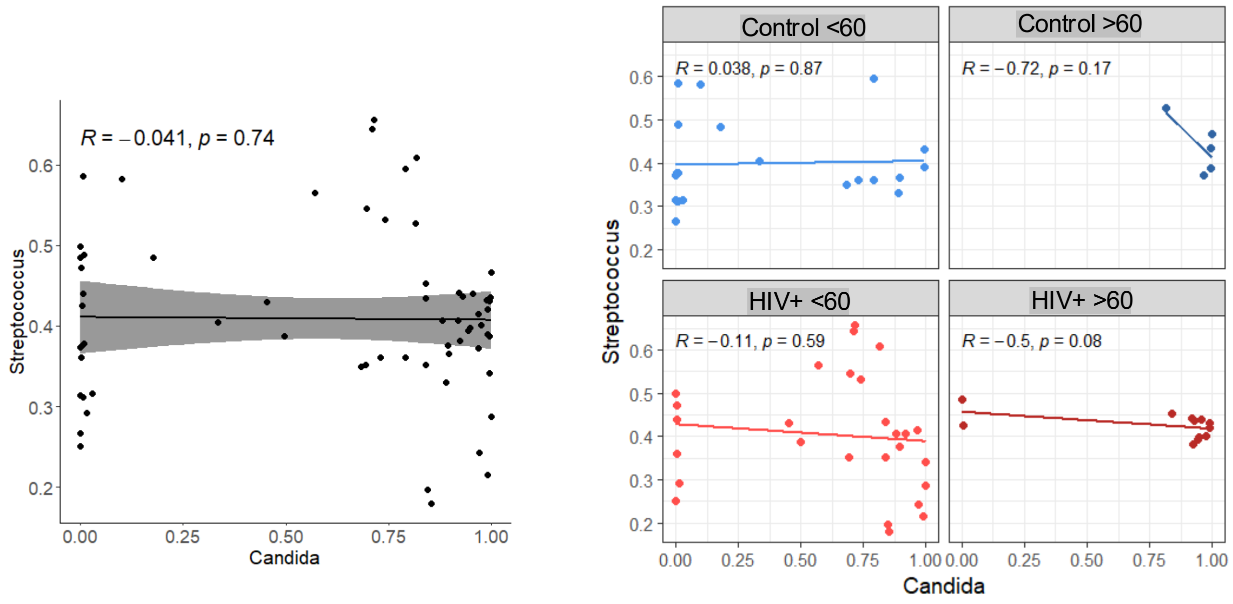

Supplementary Figure.3. Regression and Correlation analysis between *Candida* and *Streptococcus*. Scatter plot showing the association between *Candida* and *Streptococcus* relative abundance across all participants. The black line represents the fitted regression line with 95% confidence interval (gray shading). Spearman correlation was used to evaluate the relationship, yielding R=−0.041 with p=0.74. No significant association was observed, suggesting an overall weak and inconsistent relationship between the two taxa in the cohort (left) and the subgroups(right).

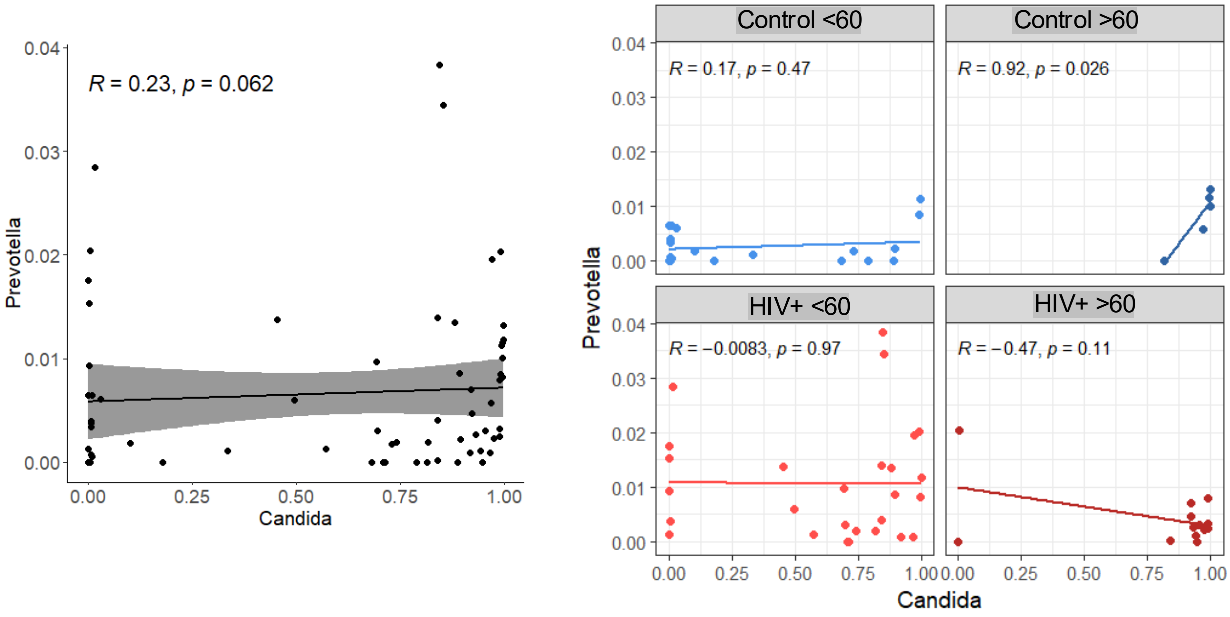

Supplementary Figure.4. Regression and Correlation analysis between *Candida* and *Prevotella*. Scatter plots showing the correlation between *Candida* and *Prevotella* relative abundance in all participants (left) and across four subgroups (right). A statistically significant moderate positive correlation was observed in the aged control group (R=0.92, p=0.026). Other subgroups showed weak and non-significant associations, suggesting that the relationship between these taxa may be context-dependent.

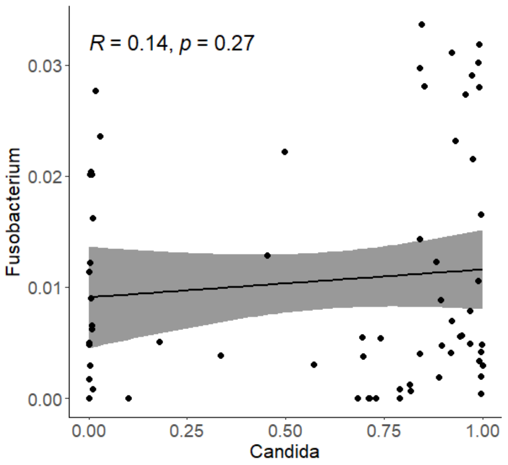

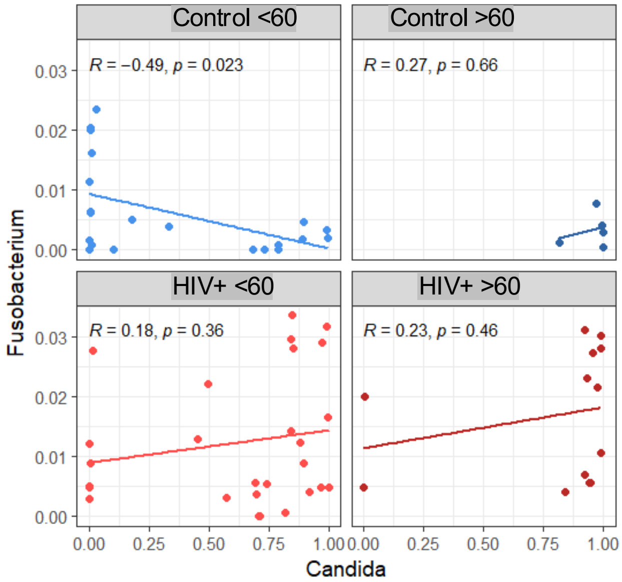

Supplementary Figure.5. Regression and Correlation analysis between *Candida* and *Fusobacterium*. Scatter plots showing the correlation between *Candida* and *Fusobacterium* relative abundance across four subgroups. Spearman correlation coefficients and p-values are displayed in each panel. A statistically significant moderate negative correlation was observed in the young control group (R=−0.49, p=0.023). Other subgroups showed weak and non-significant associations, suggesting that the relationship between these taxa may be context-dependent.
